# The social geography of fear and acceptance: an interview study on attitudes toward LGBQ identities in residential aged care

**DOI:** 10.1186/s12877-025-06234-8

**Published:** 2025-07-30

**Authors:** Klara Le

**Affiliations:** School of Behavioural, Social and Legal Sciences, Örebro University, Örebro, Sweden

**Keywords:** LGBTQ, Qualitative method, Aged care, Nursing home, Sexuality, Ageing, The social geography

## Abstract

**Background:**

Older LGBQ (lesbian, gay, bisexual and queer/questioning) adults in residential aged care (RAC) navigate environments shaped by heteronormative norms, institutional structures, and the social dynamics of communal living. Unlike in private homes, RAC settings bring together individuals with diverse backgrounds, values, and attitudes, creating both opportunities for inclusion and risks of exclusion. This study examines how both LGBQ and non-LGBQ residents contribute to shaping the social and cultural conditions of RAC and how these dynamics influence older LGBQ adults’ ability to express their sexual identity.

**Methods:**

This study employed a qualitative approach, conducting semi-structured interviews with 17 residents aged 78–95 years in Swedish RAC, including five individuals who identified within the LGBQ spectrum. A thematic reflexive analysis was conducted, drawing on Valentine’s Social geography to examine how institutional norms, peer dynamics, and spatial conditions influence residents’ opportunities to express their sexuality.

**Results:**

Findings revealed three main themes: Geography of RAC: Space and Norms, Sexual Identity and Belonging and Strategies for Safety and Visibility. The results suggests that heteronormativity and perceptions of ageing shape the attitudes within RAC environments, often rendering older LGBQ adults invisible as sexual beings. The presence and attitudes of other residents were found to play an important role in shaping the space, either reinforcing exclusion or fostering belonging. LGBQ residents described employing various strategies to navigate this terrain. While some participants experienced a sense of restriction, others found ways to assert their identity and challenge dominant norms.

**Conclusions:**

The study highlights that RAC is not only structured by formal policies but also co-created through everyday interactions among residents and staff. While previous research has emphasised the need for staff training and inclusive policies, these findings suggest that education alone may not be sufficient. This study argues that there may be value in establishing dedicated residential care facilities that are explicitly LGBTQ-friendly.

## Background

For most older adults, a move to residential aged care (RAC) represents a major transition [32]. In this new context, not only are individual needs and habits challenged, but also values and attitudes. For some, moving to RAC means the opportunity to build new communities, while for others, this new context can represent heightened exposure accompanied by a fear of not being accepted. This transition can be particularly complex for those who do not conform to normative expectations such as older lesbian, gay, bisexual and queer/questioning (LGBQ) adults [4, 37]. Despite the increased societal acceptance of LGBQ identities, studies indicate that many older LGBQ adults worry about moving to RAC, fearing the reinforcement of heteronormative norms that may pressure them into concealing their identities [17, 21]. For example, [16] found that among older GLBT (gay, lesbian, bisexual, and transgender) individuals living outside of RAC, 73.2% believed that GLBT residents in RAC face discrimination, compared to 67.7% of heterosexual respondents.

While RAC is meant to be like a home environment and a place of care and support, it can also be a place where societal biases are reproduced. A review concerning older LGBT adults revealed that many LGBT individuals feared discrimination from staff and fellow residents [22]. This perceived discrimination can lead LGBT individuals not to disclose their sexual identity or seek care altogether. Similarly, an integrative review by [9] highlights that many LGBTQ older adults fear discrimination and invisibility in RAC. This phenomenon, is sometimes referred to as heteronormative silence [28]. Heteronormative silence occurs when non-heterosexual identities are either not acknowledged or are actively denied. Research suggests that staff often approach care through a heteronormative lens, assuming all residents are heterosexual and structuring social activities, housing arrangements, and communication in ways that reinforce these assumptions [8, 30]. Many staff members also adopt a “one-size-fits-all” approach to care, claiming to treat all residents equally, regardless of sexual identity [20]. While this approach may be intended to promote equality, it often fails to recognise the specific experiences of LGBQ older adults, further contributing to their invisibility within RAC.

While much attention has been given to how staff influence care settings, an equally pressing issue is how non-LGBQ residents’ attitudes and perceptions contribute to shaping the RAC environment. Studies suggest that negative attitudes among non-LGBQ residents towards LGBQ identities can create an unwelcoming atmosphere, reinforcing feelings of exclusion among LGBQ individuals [36]. Research on the acceptance processes highlights that acceptance is often not immediate, but rather an evolving and relational process shaped by broader cultural norms. For example, [12] in a review of parental responses to young lesbian and gay individuals, emphasises that acceptance is not a static state, but an evolving relational process shaped by wider social and cultural contexts. Similarly, in RAC, everyday interactions between residents actively shape the social climate, influencing whether LGBQ individuals experience these spaces as safe or exclusionary [27].

Many older LGBQ adults have lived through decades of discrimination and entering a care setting where they are met with dismissiveness or prejudices can deepen their sense of social isolation. The lack of social recognition can have serious implications for the well-being of LGBQ older adults [11]. This aligns with the concept of minority stress, originally introduced by [2] in her work on lesbian women. The minority stress model was later developed and formalised by [23]. Minority stress refers to the psychological burden resulting from chronic exposure to discrimination and the fear of prejudice. Minority stress highlights how societal stigma and internalised negative beliefs contribute to adverse health effects [25]. Consequently, minority stress has been linked to poorer mental and physical health outcomes, including depression, anxiety, and cardiovascular disease [3, 6, 10]. If the RAC environment reinforces social exclusion, this stress can persist into later life, exacerbating health disparities between LGBQ and non-LGBQ populations. Given these challenges, it is essential not only to examine the individual experiences of older LGBQ adults but also to explore how the attitudes and behaviours of non-LGBQ residents shape the RAC environment.

It is important to understand how space, sexuality, and community interact when exploring the experiences of older LGBTQ + adults in RAC. [13] shows, in an integrative review, that migration, cultural expectations, and discrimination can strongly influence how LGBTQ + individuals feel a sense of belonging and social connection. Queer spaces, feminist groups, and pride events have long served as important places for self-expression and social connection, offering a greater sense of belonging than mainstream society [29]. In response to exclusion from traditional family structures, many older LGBQ adults have formed chosen families such as informal support networks that offer both emotional connection and practical assistance [8, 15, 26]. However, moving to RAC can feel like leaving behind these connections and entering an environment where their identities may not be recognised or valued [11].

There is growing recognition that mainstream RAC facilities often fail to meet the specific needs of LGBTQ + older adults [5]. Research in UK RACs shows that while education is an important part of recognising LGBTQ + residents, sustained organisational and cultural change is essential to creating truly inclusive environments for LGBTQ + residents [14]. In response to the challenges of promoting LGBTQ + inclusion in RAC, community-driven models of LGBTQ+-specific housing have emerged internationally, offering more affirming environments [5].

By including the perspectives of both LGBQ and non-LGBQ residents, this study aims to explore how attitudes shape the social and spatial dynamics of everyday life in RAC, and how these dynamics influence older adults’ sense of safety, visibility, and inclusion. It addresses the following questions:

- What spatial and social dynamics within RAC influence the formation and expression of attitudes toward LGBQ identities?
- How do older adults navigate these environments concerning their sexual identity?

### Social geography

This study draws on the concept of social geography. The theory provides a framework for understanding how spatial environments, together with social norms and power relations, influence the lived experiences of minority groups. This perspective can be useful when exploring how older LGBQ residents navigate RAC settings.

Social geography illustrates how certain places are constructed as accessible to some groups, while others may experience them as exclusionary or even threatening. According to [35] the ability to navigate space must be understood in relation to social relationships, identities, and power structures. These factors create and uphold boundaries within the spatial landscape, which can be physical and symbolic, affecting who has access to certain places, when, and in what manner.

Social geography has been employed in feminist geography, specifically in what [34] calls “geography of women’s fear”. This concept describes how women’s fear of male violence shapes their perception and use of public space. Women’s fear limits their freedom of movement, access to resources, and opportunities, which in turn reinforces male dominance. Women develop mental maps of risk, identifying dangerous places and times based on personal experiences, narratives, and media reports [34]. These mental maps influence their movement patterns and restrict access to public spaces.

Valentine’s theory is often used to examine how women navigate spaces perceived as unsafe [24]. For example, [24] expand Valentine’s focus on avoidance and fear by introducing the concept of “spatial confidence,” highlighting how women can reclaim spaces through repeated use or by neutralising fear. An important aspect of the social geography is that it does not solely focus on constraints but also highlights agency and resistance. Individuals can actively work to overcome their fear, for example, by “reclaiming” places they previously avoided. In the context of RAC, this theory can help identify if older adults feel excluded from shared spaces, whether they adapt their behaviour to avoid visibility, or find ways to assert their presence and identity.

### Methodology

A qualitative methodology was chosen to capture in-depth and nuanced perspectives. To facilitate this, semi-structured interviews were conducted, providing a balance between structure and flexibility [18].

### Data collection

The data were used to produce two articles: the first focused on sexuality in terms of sexual expression in general [19], while this study examines issues related to LGBQ identities, norms and attitudes. During the interviews, participants frequently returned to topics concerning LGBQ. Considering this, experiences specifically addressing these topics were extracted for this study. Only codes and themes related to LGBQ issues from the broader dataset were analysed in this study, to avoid overlap between the two publications.

To further enhance the empirical foundation, two additional interviews were conducted with LGBQ participants to deepen the understanding of their experiences in RAC. All interviews were included, even those with non-LGBQ participants. This decision was based on the understanding that all individuals contribute to shaping the conditions of the shared environment. Many participants, regardless of identity, reflected on LGBQ issues. Including these perspectives provides a more comprehensive understanding of the environment, highlighting not only how LGBQ older adults experience RAC but also how the attitudes and behaviours of non-LGBQ older adults contribute to creating conditions of safety and unsafety.

Data collection for the study was conducted within Swedish RAC from February to November 2024. A total of 40 RAC facilities were contacted and nine facilities participated in the study. In Sweden, RAC provide long-term housing and care for older adults who require daily assistance. These facilities are publicly funded and operate under a person-centred care model, aiming to support residents’ individuality, autonomy, and well-being while fostering a homelike environment. Swedish RACs typically consist of small-scale units where residents have private rooms or apartments, often with shared common areas [31, 33]. Care is provided by trained staff, and facilities commonly include specialised units for individuals living with dementia or somatic health conditions.

A recruitment package, consisting of an informational sheet and a video outlining the study’s objectives and methods, was provided to unit managers for distribution among residents. Interviews were conducted either in RAC or via telephone and lasted between 30 and 60 min. The interview followed a semi-structured guide, developed highlighting themes relevant to the study’s objectives. The thematic structure of the interview guide covered several key areas, including background information, experiences of sexuality, partnerships, and influencing factors such as interactions with staff, fellow residents, and family members. All interviews were audio-recorded with the participant’s consent and transcribed verbatim. The interviews were conducted in Swedish and subsequently translated with the assistance of a language reviewer to ensure accuracy. All participants have been assigned pseudonyms to protect their confidentiality. To minimise risk, participants were offered the option of telephone interviews, allowing those concerned about privacy to participate more comfortably.

### Participants

Participants were selected based on two primary criteria: they were required to reside in RAC and demonstrate decision-making capacity. This capacity was assessed by staff, who identified adults they deemed capable of providing informed consent. Additionally, some participants were recruited through recommendations from other participants. Participants were fully informed and made their own decisions regarding participation. This approach ensured that all participants met the study’s ethical requirements for voluntary and informed consent. The researcher did not conduct any further medical evaluations; individuals who independently reached out to participate in the interviews assessed their ability to participate in the study. To foster a comfortable interview environment, participants were given the option to have their partners present, although partners’ contributions were not included in the analytical process.

The study included 17 older adults aged between 78 and 95 years, with a mean age of 85.2 years, predominantly Swedish born, residing in Central Sweden. Participants lived in both private and municipal RACs located in medium to large urban areas, including the capital region and other major cities. The gender distribution was 10 women and 7 men. Of these, five participants (29%) identified within the LGBQ acronym. Specifically, participants identified as lesbian, gay, bisexual, and queer/questioning. Data saturation was monitored throughout the data collection process, and after 17 interviews, no new themes were emerging, with content becoming repetitive.

#### Data analysis

This study represents an analysis of previously collected material, focusing specifically on discussions related to LGBQ. The study used Braun and Clarke’s [1] six-step reflexive thematic analysis using NVivo to organise, manage and code the data. *The first phase* involved repeated readings to familiarise oneself with the material. Initial notes captured early impressions, recurring ideas, and notable statements, laying the foundation for subsequent coding. Reflections from this phase revealed how the environment of RAC influenced participants’ willingness to share. For example, while most interviews were conducted in private rooms, there were occasional interruptions from staff or other residents, which sometimes affected the flow of the conversation. In *the second phase*, meaningful segments of the data were systematically coded. By assigning brief labels to these segments, the core ideas related to the research questions were identified, allowing the data to be reduced into manageable parts. *The third phase* involved identifying themes, where codes were examined for patterns, and preliminary themes began to emerge. Similar codes were grouped into broader themes that reflected overarching ideas, providing the initial structure for deeper analysis. *In the fourth phase*, these themes were reviewed and refined to ensure coherence. Coded segments were checked with the research team to confirm their consistency with the identified themes, while overlapping or insufficiently supported themes were revised, merged, or discarded. During *the fifth phase*, themes were defined and named, capturing their essence and ensuring they represented distinct components of the data. Subthemes were developed where necessary to provide additional depth and complexity. *In the sixth step* of the analysis, the identified themes were brought together. This step connected the themes to the study’s overall purpose, showing how they fit into the bigger picture of the research. The analysis followed an abductive approach, starting with coding the results without applying a predefined theoretical framework. As the process developed, theoretical insights gradually emerged from the empirical observations. Through an iterative process of moving back and forth between the data and theoretical concepts, the theory was allowed to develop naturally, grounded in the findings [7].

Reflexivity played a central role in this analytic process. Reflexive thematic analysis is deeply embedded within qualitative paradigms that value the researcher’s active engagement with the data [1]. After each round of reading and coding, thoughts and reflections were documented in a research journal. The research journal was also used to reflect on the preunderstandings brought to the study, shaped by a professional background in social work and experience of working with older adults in RAC. These reflections were revisited throughout the analytic process to increase awareness of potential biases and ensure that interpretations remained grounded in the data. The journal entries, along with the coded data, were then shared with the research team. Team members were invited to propose alternative interpretations of the findings, fostering a collaborative and critical review process. These alternative perspectives were discussed in depth during regular meetings, allowing the analysis to benefit from diverse viewpoints and ensuring the robustness of the findings.

The final analysis resulted in three overarching themes, presented in Table 1 below. These themes reflect the central patterns identified across participants’ narratives.

**Table 1:** Overview of main themes

Main themes
Geography of RAC: Space and Norms
Sexual Identity and Belonging
Strategies for Safety and Visibility

## Results

The findings are organised into three main themes: The geography of RAC: Space and Norms, Sexual Identity and Belonging and Strategies for Safety and Visibility.

### Geography of RAC: space and norms

In the interviews, participants shared depictions of the RAC environment, highlighting its physical and social importance, which can be understood here as the geography of RAC. This geography is shaped not only by the material setting but also by social relationships, institutional power structures, and norms. This theme is presented in two subthemes: Spatial Constraints and Privacy and Heteronormativity and Everyday Interactions.

#### Spatial constraints and privacy

The RAC environment emerged as a space structured by routines and limited privacy. Participants described how physical layouts and institutional norms shaped what forms of intimacy and self-expression were possible or suppressed within these settings.

For Anders, a non-LGBQ respondent whose wife still lived in their shared home, the issue was the lack of privacy due to staff occasionally entering the room, as well as the physical limitation of small living spaces:

At the care home, it’s difficult to get any privacy. The rooms are small and the staff pop in now and then, even though they are very considerate. If we want to be alone, it’s hard to find a place where we won’t be disturbed. (Anders, M87)

Like Anders’ statement, Eleanor, an LGBQ participant, described the limitations imposed by the structure of RAC and the sense of unsafety present in shared spaces. However, unlike Anders, Eleanor speaks of feeling unsafe in public areas, offering specific examples to illustrate her experience:

One year we were sitting by the TV and [name] my favourite staff member says to us: ‘It’s Pride this week, maybe we should put up some flags.’ I felt so happy, but it quickly turned into self-hatred when two of the residents started shouting that they didn’t want that kind of nonsense here. The horrible thing is that I agreed. I nodded, and then I went back to my room. It was the first time I cried and felt completely alone during Pride. (Eleanor, W82)

Eleanor’s story illustrates that spaces intended for social interaction can paradoxically lead LGBQ individuals to feel isolated. Like Anders’s experience of physical constraints within RAC, Eleanor also perceives limitations in the environment. However, as an LGBQ individual, these shared spaces carry an added layer of unsafety, where Eleanor’s sexual identity intensifies feelings of exclusion.

#### Heteronormativity and everyday interactions

One of the most prominent perspectives was the heteronormative assumptions that shaped the space. Some older LGBQ adults reported feeling overlooked within this environment. For instance, Anna, an LGBQ participant, described how, when her wife was still alive and visited her at the RAC facility, staff and other residents often assumed they were just friends. Despite their close bond and shared history, their relationship was not acknowledged in the same way as heterosexual couples, reinforcing a sense of invisibility:

Well, the staff don’t consider that we might be a couple because we’re old and we’re two women. Many of them are rather reactionary in that sense. (Anna, W84)

Anna’s experience reflects how heteronormative assumptions are embedded in everyday interactions, where homosexual relationships are either misunderstood or dismissed, making it difficult for LGBQ individuals to feel fully recognised in their identities and relationships within RAC. Anna’s statement, “many of them are rather reactionary in that sense”, suggests a perception of staff as resistant to change and, consequently, as a potential threat to the safety for LGBQ individuals. In line with this, participants reinforced heterosexuality as the default norm in RAC. For example, Lisa, a non-LGBQ woman, reflected on the traditions she grew up with, describing how expectations around gender roles and relationships were deeply ingrained in her upbringing:

That people today speak so openly about homosexuality is something I find difficult to accept. Not only because it goes against what I learned about morality and the natural order, but also because I feel it undermines the values, I believe hold society together. (Lisa, W87)

Lisa’s statement shows how heteronormativity influences the room. The reflections illustrate how lifelong exposure to heteronormative norms can unconsciously influence attitudes, reinforcing the idea of heterosexuality as the “default” within shared living environments like RAC. This normative atmosphere was not only experienced by LGBQ participants but also noticed by some non-LGBQ residents, who at times witnessed homophobic behaviours. Elisabeth, a non-LGBQ participant shared an experience where she observed such behaviour:

One time, we had two men who were hugging each other a bit more than usual, and another man shouted something at them. It sounded like he was about to vomit. I honestly felt really sorry for them. (Elisabeth, W86)

In Elisabeth’s story, it becomes evident how negative attitudes are openly expressed within RAC, creating an environment where LGBQ individuals are particularly vulnerable. Like Elisabeth’s situation, some respondents openly admitted feeling uncomfortable around LGBQ individuals. Lisa, a non-LGBQ participant, mentioned the following:

If I see something that feels wrong… well, then I say it. For instance, I know there’s a woman who works here at the residence who’s married to another woman, and I’ve told her that I think it’s wrong. (Lisa, W87)

Lisa’s story illustrates how negative attitudes towards LGBQ individuals become embedded in the shared space of RAC. Lisa’s account also reveals the privilege of being heterosexual in this context, where voicing opinions does not carry the same consequences. This highlights how certain individuals are afforded greater freedom to exist and express themselves within the geography, while others must navigate it with caution. In contrast, some interviewees emphasised the importance of showing respect to all residents, regardless of sexual orientation. Ingrid, a non-LGBQ older adult, stated

First and foremost, it is about showing respect towards both the other residents and the staff here at the care home. There is a lot of gossip… many of us feel uncomfortable with the thought of expressing our sexuality when there are other people around, especially if it might be perceived as disruptive or unpleasant for others (Ingrid, W95).

In Ingrid’s statement, there is a clear intention to show respect to all residents. However, it also illustrates that non-LGBQ individuals may also feel uncomfortable expressing their sexuality out of concern for making others feel uneasy. This suggests that non-LGBQ residents navigate the limitations of the space. At the same time, LGBQ individuals face a double burden, facing not only the norms of ageing but also heterosexuality as the prevailing standard.

When reflecting on why certain norms exist within RAC, many respondents, both LGBQ and non-LGBQ, attributed them to generational attitudes shaped by past cultural and societal values. Carl, a LGBQ respondent, stated:

I know how homophobic this generation is. I wouldn’t want to expose anyone to that… and especially not myself. (Carl, M93)

Carl highlights the belief that the generation living in RAC today holds homophobic attitudes due to the norms they grew up with. Similarly, non-LGBQ participants confirmed that they were raised with different norms. Anita, a non-LGBQ respondent stated:

Our generation is not used to that. It is taboo and shameful to talk about. Most of us… or almost all of us like men if you are a woman and the other way around if you are a man. We are born that way. (Anita, W86)

The quotation above can be interpreted not as a simple reflection of generational understanding, but rather as a way of explaining and making sense of heteronormativity.

The geography of RAC: Space and Norms illustrates how attitudes about LGBQ individuals interact within RAC. Drawing on Valentine’s notion of *social geography*, this perspective underscores the importance of unveiling how social relationships and power imbalances shape one’s sense of belonging to a place [34]. The theme illuminates how conditions differ for LGBQ individuals compared to those who do not identify within this spectrum. For older LGBQ adult, this can involve feeling that their relationships are not taken seriously.

### Sexual identity and belonging

Participants shared many reflections on how living in RAC influenced their sense of sexual identity and belonging. This theme is presented in two subthemes: *Social Possibilities and Belonging* and *Appearance and Sexual Identity*.

#### Opportunities for belonging

Participants described a range of experiences related to social connection and inclusion in RAC. While some encountered opportunities for everyday interaction, many also spoke of feeling unwelcome, navigating between moments of invisibility and forms of exclusion. Henry, an LGBQ participant, said the following:

There are many of us who felt very worried about what it would be like when we moved into the residence, and whether we would have to change who we are (Henry, M87).

Henry’s statement indicates that participants worry about what life in RAC would entail. After the respondents moved in, one of the first issues they raised was how the new environment influenced their sense of sexuality. For example, Carl described how he had always been interested in dating other men but had never explored this part of his identity. Upon moving into RAC, Carl a LGBQ participant, reported feeling that it was now too late, leaving him unable to explore his sexuality in this new environment:

I have always had a slight curiosity about men, but it’s nothing I’ve dared to explore before. When I moved here, it felt like it was over… I would never get to explore my attraction to men. (Carl, M93)

Carl’s statement shows how relocating to RAC can serve as a symbolic threshold, suggesting that further exploration of one’s sexuality is no longer permissible. It also conveys a sense that, even before entering RAC, the individual felt unable to explore their sexuality, and that the move effectively ended any residual curiosity. When describing RAC, several LGBQ participants spoke of a sense of not being allowed to exist as an LGBQ person at RAC. Eleanor, a lesbian woman who had spent much of her life with a partner but was now divorced, explained:

A woman who likes women are lesbian but we are not allowed to exist in a place like this. I have spent my whole life avoiding places like this. Places where I don’t feel welcome, but now I have to… I have to receive care. (Eleanor, W82)

Eleanor describes a feeling of not being allowed to exist as an LGBQ person within RAC. Describing RAC as a place to avoid shows how the participant perceives their opportunities for expression to be limited, an insight that resonates with [35] argument that some individuals are granted freer spatial mobility than others.

The interviews also revealed varying conditions for experiencing safety and a sense of belonging within RAC. For non-LGBQ residents, the transition to RAC could represent an opportunity for new connections. For instance, Eric described how moving into RAC had opened new possibilities for interaction:

When I moved here, I hadn’t looked at a woman in years. But the more time I spend with those who live here, I also see possibilities. Sometimes I’ve heard they organise dances. But I’ve thought that I might go sometime to get some intimacy. (Eric, M83)

As a non-LGBQ individual, shared activities offered Eric opportunities to socialise. However, for LGBQ residents, the move could instead evoke a sense of isolation. For Henry, an LGBQ respondent, the transition led to a disconnection from his community:

No one would have accepted an 87-year-old man in a dress. My friends would have loved it, but I have no friends here. I am surrounded by the people I have avoided all my life. I never feel safe. (Henry, M87)

In contrast to Eric’s experience, Henry felt that he did not have the same opportunities to build friendships. For Henry, the co-residents are not a source of prosocial interaction but rather contribute to a sense of unsafety.

### Appearance and sexual identity

Another aspect raised was the importance of clothing in expressing one’s sexuality, mentioned by both non-LGBQ and LGBQ respondents. The interviewees explained that their appearance had always been a way to express their sexuality. However, this expression becomes limited in RAC, particularly if one relies on staff assistance for dressing. For example, Elisabeth described how her husband, who also lives in RAC but in another unit, is no longer able to dress up for her when they spend time together:

I feel sexual when I get to wear my nice dress and see the look in my husband’s eyes. But it’s as if the staff have taken away some of my husband’s sexuality when they dress him in his scruffiest clothes. He wants to look nice for me too… he wants to seduce me like he used to, but he can’t do that anymore… not when they dress him like that. (Elisabeth, W83)

What Elisabeth describes can be interpreted as a loss of sexual identity. Appearance is portrayed as playing an important role in feeling like a sexual person. Like Elisabeth, the LGBQ participants emphasised that appearance had always been important to them not only as a form of self-expression but also to maintain their LGBQ identity. However, moving into RAC often resulted in the loss of this expression, particularly for those requiring assistance from staff. Unlike Elisabeth, LGBQ participants expressed that it was not merely the sense that staff considered attractiveness unimportant, but also that staff often approached appearance with normative assumptions. For instance, Eleanor, an LGBQ participant, described how her friend who had always expressed her sexual identity through clothing could no longer do so after moving into RAC:

Some of us can no longer dress ourselves, and our expressions of sexuality are gone. The last time I saw [Name], a friend who has unfortunately passed away now, she was always what we called “butch”… she dressed more on the masculine way and always had short hair. But the last time I saw her, she had a longer, old-lady hairstyle and was wearing a blouse. [Name] had never worn a blouse and would never have wanted to be seen in something like that. She always wore a t-shirt with a vest… that was her style. (Eleanor; W82)

Eleanor’s statement highlights how power structures related to age and sexuality can influence an individual’s ability to express themselves within RAC. Across the interviews, both non-LGBQ and LGBQ participants described clothing choices and personal appearance as meaningful aspects of their identity. However, this expression often becomes restricted in RAC. For LGBQ older adults, they rely on staff for support, but they are also subjected to implicit expectations to conform to normative gendered dress codes. This aligns with [34] notion that norms around gender and sexuality are not only socially constructed but spatially enacted, meaning that spaces such as RAC are shaped by dominant cultural and social expectations.

### Strategies for safety and visibility

In the participants’ interviews, various strategies for negotiating the space emerged. These ranged from managing visibility, seeking safe spaces or asserting one’s rights.

#### Managing visibility

This was not only due to the lack of privacy but also as a way of coping with the sense of unsafety associated with being LGBQ in RAC. Anna, an LGBQ participant, noted:

I have hidden my whole life, so it’s nothing new to me. I have learned to find places and spaces where my love fits. When I had my partner, we had special gatherings and groups where we felt free to express our love. (Anna, W84)

For Anna, the need to seek out safe spaces in public environments was nothing new. While older LGBQ adults may have spent their lives navigating environments to find safety, non-LGBQ adults are now experiencing this need more acutely upon moving to RAC. Unlike Anders, Anna uses words like “hiding”, hiding was a strategy for safety. Similarly, the LGBQ participants explained that they not only physically avoided certain spaces but also concealed parts of their identity by attempting to blend into the prevailing norms of the RAC environment. Eleanor, an LGBQ participant, stated:

I no longer carry the rainbow flag with pride. I will always feel pride in who I am, but I don’t dare expose myself to anything. I have heard both homophobic and racist comments from several of the people living here. I don’t dare expose myself to hate crimes. (Eleanor, W82)

Eleanor emphasises a strategy that LGBQ individuals may adopt to cope with life in RAC. Respondents reported feeling compelled to hide their sexual identity and thus, as they put it, “return to the closet”. For Eleanor, concealment becomes a way to negotiate the environment by actively suppressing aspects of herself that could make her a target for prejudice.

#### Seeking safe spaces

One example of finding alternative spaces was shared by Anders, a non-LGBQ participant, who explained that when his wife visited, they would try to find moments within RAC where they could enjoy privacy:

We still try to hold on to it as best we can. We try to find moments where we can be close to each other, even if it’s just to hold hands or sit close together and talk. (Anders, M87)

For Anders and his wife, moving into RAC has meant a loss of privacy, requiring them to negotiate the space. This involved identifying more private moments within the facility when they knew staff would not interrupt. Like Anders’ experience, LGBQ participants also described managing the limitations of the space by seeking out safe areas.

One example related to seeking safe spaces was the importance of having people nearby who affirmed one’s LGBQ identity. Henry, a LGBQ-participant stated the following:

There are a few people I trust…for example, there is a woman, and I know she is married to another woman… I always talk to her when it comes to my partner’s move. She supports us, and it feels very reassuring. She also says that he is my husband… that means a lot to me. (Henry, M87)

Henry’s statement illustrates the importance of staff acknowledging residents’ identities and relationships. Such recognition not only affirms personal identity but also signals that their experiences and partnerships are respected within the broader care environment.

#### Asserting one’s rights

Another way in which participants navigated the environment was by asserting their rights. William, an LGBQ participant, exemplified a lifelong commitment to safeguarding personal freedoms, highlighting how vital it is for some individuals to defend the rights they have fought for, even within the constraints of communal living:

My husband used to say that we shouldn’t kiss or peck each other in front of others, but I didn’t care. I haven’t fought for my rights my entire life just to lose them when I grow older… it doesn’t work that way. (William, M86)

William describes having fought for their rights over many years and remaining unwilling to relinquish them, even after moving into an RAC. This can also be seen as an attempt to assert LGBQ older adults’ presence within RAC, challenging the heteronormative norms.

The theme *Strategies for safety and visibility* shows that both non-LGBQ and LGBQ older adults negotiate the limitations and possibilities in RAC. Upon moving into RAC, individuals are often required to adopt new strategies to manage this unfamiliar setting. However, many LGBQ individuals must navigate not only norms related to ageing and the institutional environment, but also those surrounding sexual identity and heteronormativity. These strategies are not entirely new; rather, they have been shaped over a lifetime in response to societal norms and environments that have historically overlooked or invalidated their identities.

## Discussion

This study aimed to understand how attitudes shape the social and spatial dynamics of everyday life in RAC and how they influence older adults’ sense of safety, visibility, and inclusion. It addressed two main questions: What spatial and social dynamics within RAC influence the formation and expression of attitudes toward LGBQ identities and what strategies older adults employ to navigate these environments.

The results suggest that various norms and attitudes intersect, shaping residents’ experiences. This environment, which may be described as the “geography of older LGBQ adults’ fear”, is shaped by a combination of physical, social, and cultural factors that collectively impact the sense of security and belonging for LGBQ individuals in RAC. Under the theme “Geography of RAC: Space and Norms,” both physical and social constraints within RAC are described. For example, communal areas, which are intended to serve as meeting places, were identified as unsafe spaces for LGBQ residents. This aligns with [34] concept that public spaces inherently contain limitations for certain groups. [34] argues that public environments are not neutral but are instead shaped by societal norms that can marginalise those who do not conform to dominant expectations. In this context, RAC becomes a place where heteronormative norms are reinforced, creating an environment where LGBQ individuals may feel excluded. This finding confirms previous research, that has also highlighted the persistence of heteronormativity in care environments and its limiting effects on LGBQ residents [8, 9, 30]. This study found that personal appearance was an important factor for all respondents, but for LGBQ individuals, it was particularly intertwined with their LGBQ identity. This sense of self-expression becomes restricted within RAC, indirectly pressuring individuals to conform to the prevailing norms.

While living in RAC can limit sexual opportunities for all residents, older LGBQ adults face the additional challenge of navigating both age-related and heteronormative expectations. Assumptions about older adults as asexual, combined with restrictive ideas about acceptable sexual identities, create extra barriers to expression. As a result, older LGBQ residents must negotiate not only the institutional norms of RAC but also broader societal beliefs about who is allowed to express sexuality. For many, this produces a living environment that feels both unsafe and exclusionary. This underscores that expressions of LGBQ identity in RAC are not simply personal choices, but shaped by the intersecting dynamics of age, sexuality, and institutional culture.

Unlike [34] conceptualisation of public environments, the shared spaces within RAC are not solely public but are also part of the individual’s home. This blending of public and private spheres makes it more challenging for residents to avoid these areas, leading to a complex dynamic where safety and privacy are constantly negotiated. As [34] discusses, individuals engage in mapping their surroundings to identify areas where they feel safe or at risk. In the context of RAC, this process is carried out by both non-LGBQ and LGBQ participants; however, for LGBQ individuals, the process is often more complex and heightened due to their identities. This mapping of the space reflects a heightened awareness of the social landscape and the potential risks associated with being visibly LGBQ within RAC. This aligns with findings from [13] who, in a transnational context, demonstrates how LGBTQ + individuals’ openness is shaped by spatial and cultural conditions. Like [13] this study found that self-expression often depends on whether the surrounding environment is perceived as accepting or hostile. Disclosure is not static but negotiated depending on place, safety, and anticipated reactions.

One aspect that makes RAC particularly unique is the blurring of private and public life. Individuals, many of whom may have avoided each other throughout their lives, suddenly find themselves sharing communal living spaces with individuals they may have previously avoided or with whom they may not feel safe. In line with previous research [36] this study shows that peer dynamics are a key factor for feelings of safety and unsafety, as non-LGBQ co-residents’ views on gender and sexuality sometimes express subtle or open hostility. Although some residents condemn such discrimination, the interviews suggest that negative attitudes still lead many LGBQ adults to withdraw from communal spaces or suppress aspects of their identity. This dynamic mirrors Ghosh’s (2019) findings on parental acceptance of young lesbian and gay people, where acceptance is shifting as a relational process, shaped by prevailing norms and contexts. Just as young people navigate parental acceptance within the intimate setting of the family home, LGBQ residents in RAC must navigate to whom they can safely express their identities. In both cases, safety and visibility are negotiated within everyday interactions, where acceptance remains partial, conditional, and influenced by the social climate of the shared environment.

As noted by previous research [15], the negative attitudes towards LGBQ were explained by the interviewees to be rooted in the fact that many older adults living in RAC grew up at a time when homosexuality was far less accepted. Explaining negative attitudes towards LGBQ individuals can also become a way to reinforce heteronormativity, ultimately exacerbating the marginalisation of LGBQ people. By attributing such attitudes to generational differences, there is a risk of normalising discriminatory behaviours rather than challenging them. Framing homophobic behaviours as a generational attribute can contribute to the normalisation of exclusionary attitudes and, as a result, reproduce what earlier research has described as *heteronormative silence* [28].

Like [34] describes individuals reclaiming unsafe public spaces through specific strategies, this study highlights how participants navigate their sexual identity within RAC. In [34] concept of the geography of fear, individuals are described as avoiding unsafe places at certain times of the day. However, for older LGBQ adults living in RAC, this strategy becomes more challenging as the unsafe space is also their home, and some require assistance to leave the premises. The findings reveal that individuals actively seek safe spaces to gain more privacy and visibility. However, like previous research [22], LGBQ participants also described feeling pressured to conceal their LGBQ identities to avoid discrimination. The decision to hide their identity as non-LGBQ is therefore unsurprising, given that they cannot escape the unsafe environment. Nevertheless, the LGBQ participants explained that they have developed strategies throughout their lives to conceal their identities in unsafe places.

An important factor influencing how individuals navigate life in RAC concerning their sexual identity, and thereby their “spatial confidence,” is the lack of opportunities for mobilisation and community connection. In line with previous research (Silverskog & Bromseth, 2019), LGBQ participants in this study expressed a sense of loss related to community and the ability to mobilise socially or politically. This loss is particularly important, as mobilisation and the support of like-minded groups have historically been essential for LGBQ individuals to reclaim space and build resilience. The inability to mobilise within RAC not only limits the formation of safe spaces but also impacts the individuals’ sense of agency and belonging.

In summary, this study suggests that LGBQ identities in RAC are shaped by various aspects of safety and unsafety, impacting individuals’ expressions and experiences. The findings reveal the differing conditions faced by LGBQ and non-LGBQ residents. Living in RAC can restrict an individual’s sexual opportunities, but for older LGBQ adults, it also means residing in an environment that feels unsafe despite being their home. Older LGBQ adults must contend with the combined pressures of age-related norms and heteronormative expectations. They are thus navigating not only the constraints of institutional living but also longstanding societal assumptions about who can or should express their sexuality.

By employing geography of fear [34], this study highlights how RAC settings are imbued with power dynamics reflecting broader societal norms and values. Consequently, older LGBQ adults are forced to constantly negotiate identity, safety, and acceptance. Considering these findings, this study highlights the limitations of the “one size fits all” approach to care, as discussed in earlier research [8, 20, 30]. This approach risks overlooking the unique challenges faced by older LGBQ individuals living in RAC, thereby failing to address their specific needs and experiences. Creating genuinely inclusive care environments requires not only structural changes such as reconsidering the organisation of communal spaces but also sustained cultural shifts that challenge heteronormative assumptions.

Although non-LGBQ older adults also navigate identity and spatial factors, this study shows that those with LGBQ identities encounter additional layers of complexity. Using the concepts of safety and unsafety proves valuable, as a persistent sense of unsafety can have negative health implications, including heightened stress, anxiety, and isolation. Living with minority stress, where chronic exposure to discrimination and fear of prejudice can have serious impacts on mental and physical well-being. For older LGBQ adults, these challenges are compounded by a lifetime of navigating public spaces perceived as unsafe, fostering an ongoing sense of minority stress. This stress now follows them into RAC, which is supposed to be a safe home environment. The institutional context may also bring back earlier experiences of exclusion, making daily interactions with co-residents and staff a new source of stress. In this way, minority stress becomes not just an internal experience but something that is reinforced in the shared space of RAC.

Given the study’s findings, it is not surprising that many LGBQ individuals express anxiety about their future within RAC. This concern aligns with previous research showing that LGBTQ older adults often experience fear of discrimination and anticipate having to conceal their identities in long-term care settings, contributing to feelings of anxiety and uncertainty about future care [9]. The emergence of LGBTQ-specific housing (Concannon, 2022) can be seen not only as a response to these concerns but also as an indication of the persistent structural and cultural limitations of mainstream RAC in meeting the needs of LGBTQ residents.

This study adds to the growing body of research on sexual minority older adults in care settings by empirically demonstrating how co-residents play an active role in shaping the spatial and normative conditions of RAC. In line with [36] who identified the presence of negative attitudes among some Spanish residents, this study moves further by adopting a dual-perspective approach, underscoring how peer dynamics in RAC co-create the conditions for belonging, visibility, and participation.

Limitation.

A primary limitation is that this study does not encompass the full diversity represented by the LGBTQI + acronym, omitting those who have trans experience, an intersex variation or belong to other identities. Consequently, experiences unique to these communities remain unaddressed. Future research should aim to include these diverse identities to gain a more comprehensive understanding of the challenges and experiences faced by all members of the LGBTQI + community. Another constraint concerns the relatively small number of participants who identified as LGBQ. Recruitment was challenging in part because many older LGBQ adults in RAC feel uncomfortable disclosing their identities, fearing potential discrimination. To mitigate these concerns, interviews were conducted by telephone, offering participants a degree of anonymity and reducing barriers to involvement.

## Conclusion

The findings of this study indicate that interactions within RAC play an important role in shaping institutional culture, highlighting that older adults themselves actively contribute to either maintaining or challenging heteronormativity. By integrating both LGBQ and non-LGBQ perspectives, the study demonstrates how conditions within RAC differ between these groups. While non-LGBQ older adults often align more seamlessly with the dominant norms, older LGBQ adults frequently encounter environments perceived as unsafe, which may make them feel compelled to conceal parts of their identity.

Furthermore, this study challenges the assumption that increased staff training and cultural competence alone can foster inclusive environments. Instead, it underscores the importance of recognising the collective influence of residents in shaping the social climate within RAC. Therefore, fostering inclusivity may require not only educational initiatives but also the development of LGBTQ-friendly residential care options that ensure safety, acceptance, and a sense of community for older LGBQ adults.

## Data Availability

For reasons of confidentiality, the data is not made public. Pseudonymised data in Swedish is available upon request from the author.

## Declarations

Use of AI: AI has been used in this study primarily to review and enhance the language used in the text. It has been employed to find suitable synonyms and improve sentence structure.
